# Effects and Safety of 5% Lifitegrast Ophthalmic Solution in Patients With Dry Eye Disease Associated With Ocular Graft-Versus-Host Disease

**DOI:** 10.7759/cureus.66437

**Published:** 2024-08-08

**Authors:** Calvin W Wong, Harrison L Le, Dan S Gombos, Richard W Yee

**Affiliations:** 1 Ophthalmology, McGovern Medical School, University of Texas Health Science Center at Houston, Houston, USA; 2 Ophthalmology, Richard W. Yee, MD PLLC, Houston, USA; 3 Ophthalmology, The University of Texas MD Anderson Cancer Center, Houston, USA

**Keywords:** ocular graft versus host disease, dry eye, graft versus host, xiidra, lifitegrast

## Abstract

Introduction

Graft-versus-host disease (GVHD) is a common sequela of hematopoietic stem cell transplant (HSCT). While HSCT is often curative for certain hematologic malignancies, acute and chronic GVHD remains an important cause of morbidity and mortality in post-transplant patients. Ocular involvement is one manifestation of chronic GVHD that can present similarly to chronic dry eye with tear film abnormalities, aqueous deficiency, and corneal epithelial defects through melting and perforation. Current management includes frequent use of artificial tears and oral or topical glucocorticoids as tolerated. There is a need for long-term, steroid-sparing therapeutics in the management of ocular GVHD (oGVHD). Lifitegrast is approved for the treatment of chronic dry eye and may have therapeutic potential in the treatment of oGVHD. The aim of this study was to investigate the efficacy and safety of topical lifitegrast in the management of oGVHD.

Methodology

A prospective randomized clinical trial (NCT04792580) was performed on 32 enrolled patients with diagnosed oGVHD. Subjects underwent a two-week washout period consisting of preservative-free artificial tears dosed twice a day, after which they were randomized to the treatment arm (5% lifitegrast ophthalmic solution) or placebo arm (vehicle solution) for four weeks. Endpoints included Symptom Assessment iN Dry Eye (SANDE) score, unanesthetized Schirmer score, Ocular Surface Disease Index questionnaire score, fluorescein staining, tear film breakup time, meibum quantity, and turbidity. Safety endpoints included intraocular pressure, visual acuity, and rate of treatment-related adverse effects. Statistical analysis was done with a t-test or Wilcoxon rank-sum test.

Results

The primary and secondary efficacy endpoints were met, with statistically significant reductions in mean SANDE and unanesthetized Schirmer score observed at four weeks post-randomization. No serious adverse events related to the use of either lifitegrast or vehicle were observed, and no worsening of visual acuity or intraocular pressure occurred in the intention-to-treat analysis. However, further inference was limited due to insufficient statistical power owing to significant washout and a 50% dropout rate from the all-enrolled analysis set. The most common causes of study dropout were worsening of unrelated medical conditions (not GVHD) and improvement of SANDE score or Schirmer score outside of the inclusion criteria range during the washout period.

Conclusions

Lifitegrast may be a useful steroid-sparing agent in the long-term management of oGVHD. This study provides further support for the clinical evidence of lifitegrast in the management of dry eye signs and symptoms, although further sufficiently powered clinical trials are warranted to better understand its efficacy in the oGVHD population. Personalized treatment options targeting distinct manifestations of oGVHD in the cornea, tear film, lid margin, and conjunctiva are needed in the effective management of this multifaceted and complex disease.

## Introduction

Graft-versus-host disease (GVHD) is a common complication of hematopoietic stem cell transplant, most commonly for hematologic malignancy. Despite recent advances in donor matching, prophylaxis against GVHD, and transplant technology, GVHD remains a significant cause of morbidity for these chronically immunosuppressed patients. Acute GVHD classically develops three or four weeks post-transplant, while chronic GVHD usually develops after 100 days. Further, the pathophysiologies of chronic and acute GVHD are biologically distinct from each other: acute GVHD is a result of cytokine-related mediated by donor T cells and host tissue reactivity, and chronic GVHD is related to dysthymic function, development of autoreactive T cells, and upregulation of proinflammatory cytokines [1-3]. While cutaneous, hepatic, and gastrointestinal involvement are more common in acute GVHD, a distinct manifestation in chronic GVHD is ocular involvement.

Due to the distinct pathophysiologies of acute and chronic GVHD, we have chosen to study ocular involvement in chronic GVHD exclusively. The development of ocular GVHD (oGVHD) is thought to be related to inflammatory and lymphocyte-mediated cytotoxicity impairing lacrimal function, leading to aqueous tear film abnormalities characteristic of dry eye syndrome. oGVHD is therefore analogous to Sjögren’s syndrome and presents with similar clinical findings in the mucosa [4,5]. Specifically, scleroderma-like changes to the eyelids, entropion, and lagophthalmos may be present, while corneal findings may include punctate or diffuse keratitis, ulceration, melting, or if left untreated, perforation. Posterior segment findings of oGVHD include lipid deposits, retinal or vitreous hemorrhage, microangiopathy, and optic disc edema [6,7]. Tear production is often abnormal, with either excessive reflex tearing or insufficient tear production as measured by the Schirmer tear test.

Current treatment of oGVHD is often identical to conventional dry eye therapeutics in addition to systemic immunosuppressants like steroids, tacrolimus, or sirolimus. These conventional treatments include warm compresses, artificial tears, and in more severe cases topical corticosteroids. Often, such treatments are inadvisable or insufficient to prevent the development of more severe ocular manifestations. Corneal findings may include ulceration, melting, or even perforation, while the tear film may show evidence of aqueous deficiency, meibomian gland dysfunction, and instability as measured by breakup time. Conjunctival involvement may manifest as pseudomembranous conjunctivitis or symblepharon. In these cases, glucocorticoids may prove temporarily useful, but their long-term use is limited by the development of cataracts and elevated IOP. In the absence of other advisable therapeutics, tarsorrhaphy or scleral contact lenses are recommended.

oGVHD can therefore be debilitating for patients with a history of stem cell transplant, and an early diagnosis of oGVHD versus chronic dry eye is important in guiding aggressive treatment. There is a need for glucocorticoid-sparing therapeutics targeting novel mechanisms in the management of oGVHD, and recent studies have investigated the use of treatments such as topical spironolactone, progesterone, and heparin [8-10]. However, larger prospective studies of therapeutics in oGVHD are rare.

Lifitegrast ophthalmic solution, 5%, has been approved by the United States Food and Drug Administration for managing signs and symptoms of dry eye disease. Lifitegrast is a competitive antagonist for the lymphocyte function-associated antigen 1 (LFA-1), which mediates T-cell activation differentiation, and upregulation of proinflammatory cytokines by interaction with members of the intercellular adhesion molecule (ICAM) family [11,12]. Based on its immunomodulatory effect and its efficacy in the treatment of dry eye disease uncomplicated by GVHD, the use of lifitegrast ophthalmic solution may be beneficial in the management of signs and symptoms of oGVHD.

Treatment of oGVHD can be complicated and difficult due to the multifactorial nature of the disease. Thus, patients often fail to achieve symptomatic control despite the use of currently available treatment strategies. The aim of this study was to investigate the clinical efficacy and safety of a 5% lifitegrast ophthalmic solution in the management of oGVHD.

## Materials and methods

The present study was an investigator-initiated single-center, randomized parallel-group, double-blind, prospective study of patients with diagnosed oGVHD.

**Figure 1:**
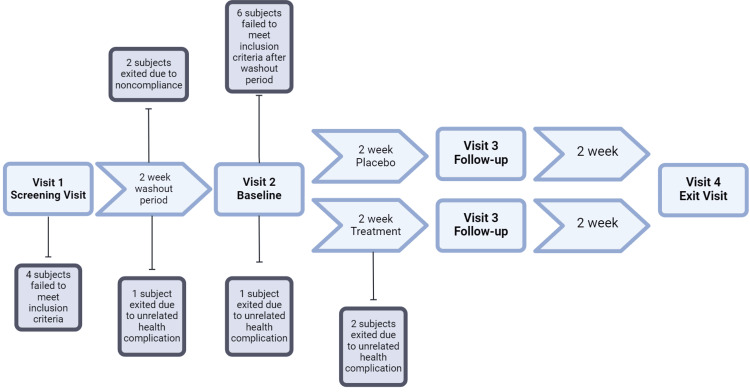
Study Schematic of Treatment Arms, Visit Timeline, and Subject Dropout

The study schedule was based on the design of the Phase III OPUS-2 study [13]. The schedule consisted of a two-week washout, followed by randomization into a four-week parallel-arm period. The washout period was designed to simulate standard of care, with treatment consisting of preservative-free artificial tears (Systane® PF; Alcon, Inc., Geneva, Switzerland) dosed twice a day (BID) in both eyes (OU; oculus uterque). After two weeks of washout, subjects were re-evaluated, and those meeting inclusion criteria were randomized in a 1:1 ratio to treatment (5% lifitegrast ophthalmic solution BID) and control (vehicle solution BID) groups. At two and four weeks after randomization, patients were reassessed for all study efficacy and safety endpoints with a full ophthalmic exam (Figure 1).

The primary efficacy endpoint was the change in Symptom Assessment iN Dry Eye (SANDE) score from the time of randomization to four weeks [14]. The secondary efficacy endpoint was the change in the Schirmer I test score (in millimeters) from the baseline to the last visit. Exploratory efficacy endpoints included change in lissamine green staining (Oxford schema; graded 0-5, temporally, centrally, and nasally); change in tear film breakup time (TFBUT); change in Ocular Surface Disease Index (OSDI) score; change in bulbar conjunctival injection (graded 0-4); change in palpebral conjunctival redness injection (PCR; graded 0-100 analogous to Validated Bulbar Redness scale); and change in corneal fluorescein staining (CFS; National Eye Institute schema, graded 0-3, temporally, centrally, nasally, superiorly, and inferiorly) [15-17]. The same investigator graded each of the above measures.

Safety endpoints included rate of adverse events (AEs), serious AEs, after the first randomization dose of the investigational product; changes in best-corrected visual acuity (BCVA), tolerability to eye drops at each visit (graded 0-100 on the visual analogue score (VAS)).

Subject inclusion criteria, assessed at screening and randomization visit, consisted of the following: diagnosis of chronic GVHD; “Probable” or “Definite” oGVHD [18]; baseline SANDE score of >40; Schirmer I score between 2 mm and 10 mm. Exclusion criteria were ocular infection, systemic or ocular allergies, intraocular inflammation, systemic disease not stabilized within one month before screening, pregnancy, contact lens or punctual plug use 30 days prior to or during the study, use of topical cyclosporine, corticosteroid, or other dry eye treatment 30 days prior to or during the study, prior ocular or adnexal surgery 90 days prior to first visit, previous use of lifitegrast ophthalmic solution, 5%, or use of the rescue treatment. Eyes that met all inclusion criteria were qualified as the study eye. If both eyes qualified as the study eye, the right eye was selected as the study eye.

Randomization, descriptive statistics, and statistical analyses were performed using STATA 16 and 17® (StataCorp LLC, College Station, Texas, USA). A block randomization list was generated, and primary investigators were blinded to block size to minimize the risk of unblinding the allocation order. The randomization list was provided to a designated non-blinded, non-clinical team member. For each primary, secondary, and exploratory endpoint, a difference score from baseline at the time of randomization to week 4 was calculated. Examination of data included calculation of summary statistics such as sample size, mean (standard deviation), median (interquartile range), minimum, and maximum for continuous and discrete variables. Frequency (percentage) was calculated for categorical variables. Inferential statistical analysis was done with the Student’s t-test. For measures with non-normal distribution, the Wilcoxon rank-sum test was used.

Analysis sets included the following: all-enrolled, intent-to-treat (ITT), modified intent-to-treat (mITT), and per-protocol (PP) sets. The all-enrolled set included all subjects who signed the study informed consent and were assigned a study subject identification number. ITT set included all subjects randomized into a study treatment group. mITT set included ITT subjects who received at least one study eye drop and had efficacy assessments at both baseline and at least one follow-up visit; this was the primary efficacy set for all efficacy endpoints. The PP population was a subset of mITT subjects with no major protocol violation or deviation and with no missing primary efficacy data.

Subjects were recruited from a dry eye practice in a major metropolitan area. At all times during the study, rescue treatment, designated as use of preservative-free artificial use more frequently than BID, was available. Subjects were educated on the availability of rescue treatment, other available treatment options, and subsequent exit from the study in the case of rescue treatment usage. Subjects were trained and observed to use the correct technique in eyedrop self-administration and provided with dosing logs to confirm treatment adherence. Informed consent was obtained from each participating subject, and investigational procedures, data acquisition, and data management were done under Institutional Review Board approval with adherence to the tenets of the Declaration of Helsinki.

## Results

With a target sample size of 30, 32 subjects were enrolled. Post enrollment, four subjects failed to meet inclusion criteria. Six subjects were washed out during the first two weeks and did not meet the inclusion criteria at the randomization stage. Two subjects exited the study early due to treatment nonadherence. An additional four subjects exited the study due to health complications unrelated to the treatment. Thus, 16 patients remained in the mITT and PP sets. Ten belonged to the treatment arm, and six belonged to the placebo arm. A summary of the dropout rate and reason may be found in Figure 1.

Regarding demographics, the mITT set had a mean age of 59 (±12.8; median 63.5; range 25 to 74). The most common self-reported race in the mITT set was White/Caucasian (68.75%; n=11), followed by Asian (18.75%; n=3) and Black (12.5%; n=2). Twenty-five percent of the mITT set self-identified as Hispanic/Latino. The three most common historical malignancies in the mITT set were acute myelogenous leukemia (AML; 56.25%; n=9), chronic myelogenous leukemia (12.5%; n=2), and primary myelofibrosis (12.5%; n=2). The all-enrolled set had a mean age of 58.68 (±13.6; median 63.5; range 25 to 74). Self-reported race of the all-enrolled set consisted of White (75%; n=24), Black (12.5%; n=4), and Asian (12.5%; n=4). Twenty-five percent (n=8) of subjects self-identified as Hispanic/Latino. The most common historical malignancies were AML (40.63%; n=3), myelodysplastic syndrome (25.01%; n=8), primary myelofibrosis (9.38%; n=3), and acute lymphoblastic leukemia (9.38%; n=3).

The primary efficacy endpoint, mean change in SANDE score across the four weeks from randomization to the last visit, was achieved, with a mean reduction of 26 mm (±27 mm) in the treatment arm compared to a mean increase of 3.2 mm (±24.7 mm) in the placebo arm (p<0.05). The secondary efficacy endpoint, change in Schirmer I score (mm), was non-normally distributed, requiring statistical analysis with the Wilcoxon rank sum test. The median change in Schirmer I score was +0.5 mm in the treatment arm, compared with -3 mm in the placebo arm (p<0.05).

Examination lid margin findings, including mean lid margin vascularity (V), meibomian gland obstruction (O), meibum turbidity (T), Zone A (ZA), conjunctival injection score, TFBUT, and PCR did not show statistically significant differences by treatment arm [19]. OSDI score was not significantly different by treatment arm.

Regarding CFS and conjunctival staining with lissamine green, there were no statistically significant differences across the central, superior, inferior, and nasal zones. There was a statistically significant reduction in lissamine green staining of the temporal conjunctival in the treatment arm (p<0.05). A summary of inferential statistical analysis of the above study endpoints is provided in Table 1.

**Table 1:** Summary of Inferential Statistical Testing of Endpoints * p<0.05 ^a ^Statistical testing done with paired t-test. ^b ^Statistical testing done with Wilcoxon rank-sum test due to non-normal distribution. Reported values are medians. SANDE: Symptom Assessment iN Dry Eye; LogMAR BCVA: logarithm of the minimum angle of resolution best corrected visual acuity; OSDI: ocular surface disease index

Endpoint	Placebo Mean (± SD)	Treatment Mean (±SD)	Effect Size	p-value
SANDE score (mm)^a^	+3.2 (±24.7)	-26.0 (±27.2)	-15.0	0.049*
Schirmer test score (mm)^b^	-3	+0.5	+3.50	0.014*
LogMAR BCVA^a^	+.05 (±0.122)	+.04 (±0.171)	+.043	0.903
OSDI (0-100)^a^	+5.5 (±13.0)	-13.7 (±26.5)	-6.47	0.123
Vascularity (0-4)^a^	0 (±0)	-.1 (±0.568)	-0.063	0.677
Meibomian gland obstruction (0-4)^a^	0 (±0)	-.1 (±0.316)	-0.063	0.458
Meibum turbidity (0-4)^a^	0 (±0)	-0.5 (±0.850)	-0.313	0.177
Zone A (0-4)^a^	0.17 (±0.408)	-0.2 (±0.789)	-0.063	0.313
Tear film breakup time (seconds)^a^	-0.67 (±1.86)	-0.50 (±1.18)	-0.167	0.828
Conjunctival injection (0-4)^a^	-0.33 (±0.816)	-0.3 (±0.483)	-0.033	0.919
Palpebral conjunctival redness (0-100)^a^	-3.33 (±15.1)	-3 (±11.6)	-0.333	0.961
Corneal Fluorescein Staining (CFS), Superior (0-4)^a^	-0.167 (±0.753)	-0.100 (±0.738)	-0.067	0.865
CFS, Inferior (0-4)^a^	0 (±0.632)	+0.100 (±0.738)	-0.100	0.787
CFS, Nasal (0-4)^a^	+0.167 (±0.408)	+0.100 (±0.843)	+0.067	0.843
CFS, Temporal (0-4)^a^	+0.167 (±0.408)	-0.100 (±0.876)	+0.267	0.499
CFS, Central (0-4)^a^	0 (±0)	+0.600 (±1.17)	-0.600	0.237
Lissamine Green Staining (LGS), Temporal (0-4)^a^	+0.333 (±0.516)	-0.600 (±0.966)	0.933	0.048*
LGS, Central (0-4)^a^	0 (±0.632)	0 (±1.25)	0	1.00
LGS, Nasal (0-4)^a^	+0.167 (±0.753)	-0.500 (±0.707)	+0.667	0.096

Regarding safety outcomes, one patient experienced a corneal ulcer during the washout period, requiring exit from the study. No other serious AEs occurred. The Logarithm of Minimum Angle of Resolution (LogMAR) BCVA was not significantly different by treatment arm. There were no excursions of IOP outside of the normal range, and the mean IOP was not significantly different by treatment arm.

## Discussion

The use of lifitegrast in chronic dry eye disease has been proven in multiple clinical trials with much larger sample sizes, and achievement of the primary and secondary efficacy endpoints in this study despite significant dropout rates is suggestive of continued efficacy in oGVHD. However, this study is not without significant limitations, and further inference is limited due to insufficient statistical power.

While the study’s target enrollment was met with 32 subjects enrolled, the mITT analysis set ultimately consisted of 16 subjects, representing a 50% dropout rate. It should be noted that 6 of the 16 subjects dropped out due to improvements in Schirmer I or SANDE scores outside of the inclusion criterion range during the washout period. The significant rate of study dropout due to washout using preservative-free artificial tears BID highlights the continued importance of ocular surface lubrication in oGVHD, where treatments such as hourly artificial tear use are the first line. Additionally, the use of a Schirmer I score of 10 mm or less as an inclusion criterion resulted in the exclusion of any patients with normal or reflex tearing. Thus, it should be noted that the study sample is only representative of patients with oGVHD resistant to artificial tear usage. Further study should include not only larger sample sizes but also patients with a wider range of clinical signs and symptoms for optimal study external validity and generalization. Additionally, the use of the Schirmer I score as an inclusion criterion should be weighed against the possibility of bias when it is also used as an endpoint.

Future protocol development in the field of oGVHD should be cognizant of the risk of not only washout but also drop out. Patients with chronic GVHD necessarily carry more disease burden and comorbidity than those with chronic dry eye uncomplicated by history of hematologic malignancy and possible chemo/radiotherapy. Thus, changes in systemic disease management as well as complications due to other medical conditions are expected to be present in higher proportion relative to the chronic dry eye population. Clinical trial development for oGVHD as well as rare ocular conditions in general should therefore balance concerns regarding both internal and external study validity, especially when achieving sufficient sample sizes for optimally powered studies. The natural history of oGVHD does not exist in a vacuum, and clinical management of oGVHD with or without adjunct use of lifitegrast should anticipate possible complications such as changes in systemic medical management, infection, or exacerbation of GVHD or other comorbidity. Thus, studies with less restrictive inclusion criteria may offer greater generalizability and utility for clinical management.

Of note, the SANDE and OSDI questionnaires have been validated with moderate to excellent reliability and correlation, and while in this study there was a statistically significant reduction in mean SANDE score in the treatment arm versus placebo, there was a nonsignificant reduction in mean OSDI score (p=0.123) [20,21]. Further comparison of the use of the SANDE versus the OSDI questionnaires in clinical trials would provide valuable insight into the reliability and utility of both instruments, especially in small sample-size studies.

Clinicians should be aware of the need for recognition of oGVHD as a distinct clinical entity from severe chronic dry eye. Although frequent artificial tear use and topical glucocorticoids are most commonly employed, chronic glucocorticoid use for this chronic disease process is limited by the glucocorticoid side effect profile. To the limitations of this study, lifitegrast appears to be a viable long-term adjunct or monotherapy in the treatment of mild to moderate oGVHD, as previous studies of lifitegrast in chronic dry eye demonstrated an acceptable long-term side effect profile. Cost permitting, the use of prophylactic lifitegrast after bone marrow transplant but prior to the onset of ocular symptoms also merits consideration.

## Conclusions

Overall, the primary and secondary endpoints of this randomized control trial were achieved, as evidenced by reductions in mean SANDE and Schirmer I score from baseline to 28 days in the treatment versus the placebo arm. However, further generalization is limited due to limited statistical power. Despite the small sample size, lifitegrast continues to demonstrate a benign side effect profile, with no treatment-emergent adverse effects, worsening of vision, or IOP observed in the treatment arm. This study’s data adds to the clinical evidence supporting the safety and efficacy of lifitegrast, though larger, more inclusive clinical trials are warranted to better understand its efficacy and safety profile in the GVHD population. Lifitegrast continues to show promising clinical utility as a steroid-sparing agent in the management of symptomatic oGVHD.
